# A case of anterior cranial fossa dural arteriovenous fistula concomitant with moyamoya disease

**DOI:** 10.1016/j.radcr.2026.06.109

**Published:** 2026-07-20

**Authors:** Junki Youda, Tatsuya Shimizu, Ryousuke Shintoku, Masanori Aihara, Masahiko Tosaka, Soichi Oya

**Affiliations:** Department of Neurosurgery, Gunma University Graduate School of Medicine, Maebashi, Gunma, Japan

**Keywords:** Anterior cranial fossa dural arteriovenous fistula, Moyamoya disease, Hemorrhage, RNF213, East Asian

## Abstract

Moyamoya disease is rarely complicated by other cerebrovascular diseases. A man in his 60s presented with subcortical hemorrhage in the left frontal lobe with an anterior cranial fossa dural arteriovenous fistula (dAVF) and concomitant asymptomatic moyamoya disease. Although the anterior cranial fossa dAVF and moyamoya disease have similar vascular architectures of intra and extracranial vascular connections via the ethmoidal artery, the former involves anastomoses between arteries and veins and the latter involves anastomoses between arteries. Both diseases are rare, with our search of the literature identifying 11 cases of dAVF concomitant with moyamoya disease. These cases are reviewed. It is important to be aware that dAVF can develop during the course of moyamoya disease, and careful follow-up may therefore be warranted.

## Introduction

Anterior cranial fossa dural arteriovenous fistula (dAVF) forms a shunt from the ethmoidal artery to the cortical vein of the frontal lobe without involving the venous sinus, and frequently presents with hemorrhage [1,2]. On the other hand, advanced-stage moyamoya disease often develops transdural anastomoses from the ethmoidal artery to the anterior cerebral artery territory [3]. Although these pathologies show similar vascular architecture, the former involves anastomoses between arteries and veins, while the latter is an anastomosis between arteries. Both diseases are rare and few reports have documented the concomitant appearance of 2 [4,5]. We present a case of anterior cranial fossa dAVF concomitant with asymptomatic moyamoya disease, and discuss the case with reference to the literature.

## Case presentation

A 62-year-old man visited his local physician with a 2-day history of persistent headache and nausea. Neurological examination revealed no focal deficits. Plain computed tomography (CT) of the head showed subarachnoid hemorrhage and subcortical hemorrhage predominantly in the left frontal lobe (Fig. 1A), and he was transferred to our hospital under the careful management of blood pressure. Three-dimensional CT angiography showed dilated cortical veins with varix extending from the left olfactory fossa to adjacent to the hematoma (Fig. 1B). Cerebral angiography of the left external carotid artery showed a cortical vein with a varix draining into the superior sagittal sinus via the facial and ethmoidal arteries, forming a shunt at the olfactory fossa (Fig. 2A-C). We diagnosed anterior cranial fossa dAVF, with the bleeding point considered to be the varix. Right internal carotid angiography also showed severe stenosis of the middle cerebral artery with the developed moyamoya vessels (Fig. 2D and F). Left internal carotid angiography showed stenosis from the terminal portion of the internal carotid artery to the M1 and A1 segments, with the development of moyamoya vessels in the surrounding area (Fig. 2E). The patient had vascular risk factors associated with atherosclerosis, including hypertension and diabetes mellitus. However, thin-slice T2-weighted MRI demonstrated shrinkage of the outer vessel diameter in the bilateral middle cerebral arteries and the terminal portion of the right internal carotid artery, findings that have been regarded as characteristic of moyamoya disease (Fig. 2G) [6,7]. No other cause of arterial stenosis, such as vasculitis, was identified. Furthermore, the patient had no known conditions associated with moyamoya syndrome, including neurofibromatosis type 1, Down syndrome, thyroid disease, or prior cranial irradiation. Accordingly, the patient fulfilled the current Japanese diagnostic criteria for moyamoya disease rather than moyamoya syndrome [7,8]. Concomitant adult moyamoya disease was therefore diagnosed. No prior clinical episodes suggestive of transient ischemic attack (TIA) or imaging findings indicative of cerebral ischemia were identified. On hospital day 2, disconnection of the draining vein with unilateral left frontal craniotomy was performed to prevent rebleeding. Endovascular treatment was not selected because of the technical difficulty of catheter access, the low curative rate, and the potential risk of inadvertent embolic material migration into the ophthalmic artery. Care was taken to avoid intraoperative hypotension and hypocapnia in discussion with the anesthesiologist. Cerebral angiography on postoperative day 12 showed obliteration of shunt flow (Fig. 3). Because the patient was asymptomatic with respect to moyamoya disease and no old ischemic lesions were detected on neuroimaging, conservative observation was chosen. Genetic testing for RNF213 mutations was not performed. One month postoperatively the patient was discharged without any neurological deficit. As of 3 years postoperatively, no recurrence of dAVF has been seen and moyamoya disease has remained asymptomatic. Magnetic resonance angiography at follow-up revealed no interval changes, including no progression of arterial stenosis or development of new collateral channels related to craniotomy site (Fig. 4).

**Fig. 1:**
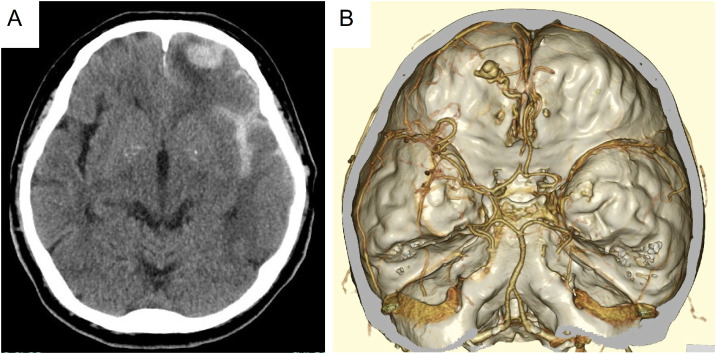
(A) Noncontrast CT demonstrates a subcortical hemorrhage in the left frontal lobe with associated subarachnoid hemorrhage extending into the left sylvian fissure. (B) CT angiography reveals a cortical vein arising from the olfactory fossa of the left anterior cranial fossa, forming a varix adjacent to the hematoma.

**Fig. 2:**
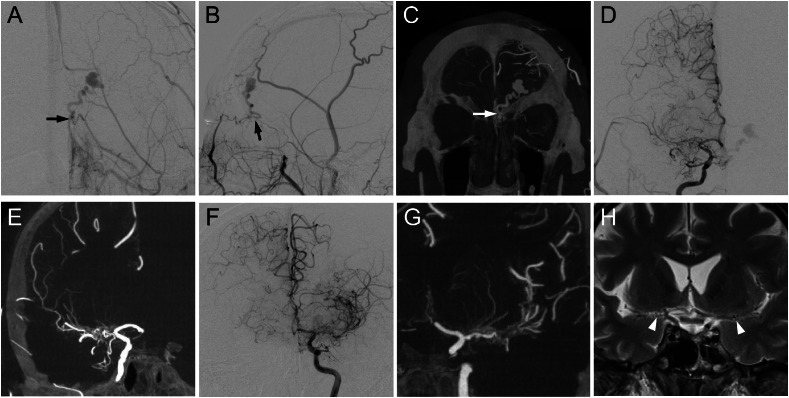
Left external carotid angiography demonstrates an anterior cranial fossa dural arteriovenous fistula supplied by the facial and ethmoidal arteries, draining into a tortuous cortical vein with a varix that ultimately empties into the superior sagittal sinus. Arrow indicates the shunt point. (A) Anteroposterior view of left external carotid angiography. (B) Lateral view of left external carotid angiography. (C) Maximum intensity projection image of left external carotid angiography. (D and E) The right internal carotid angiography shows occlusion of the middle cerebral artery (MCA) with moyamoya vessels; the MCA territory is supplied via leptomeningeal anastomoses from the anterior cerebral artery (D), and their maximum intensity projection image shows developed moyamoya vessels clearly (E). (F and G) The left internal carotid angiography also shows severe stenosis of the MCA and anterior cerebral artery with prominent moyamoya vessels. (H) Thin-slice T2-weighted MRI demonstrating shrinkage of the outer vessel diameter of the bilateral middle cerebral arteries (arrowheads), a finding consistent with moyamoya disease.

**Fig. 3:**
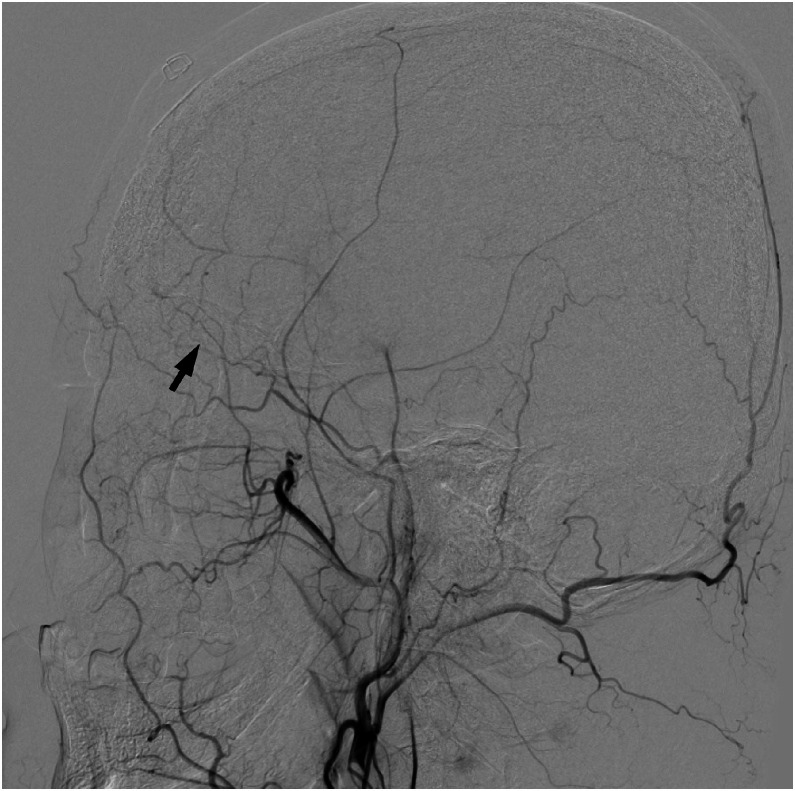
Postoperative left external carotid angiography demonstrates complete obliteration of the shunt (arrow indicates the surgical clip).

**Fig. 4:**
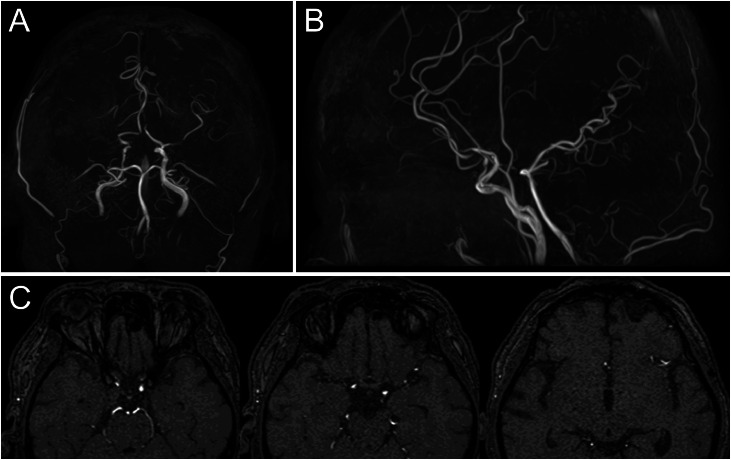
Follow-up MRI obtained 3 years after surgery. No progression of moyamoya disease or recurrence of the dural arteriovenous fistula is observed. Magnetic resonance angiography, including the anteroposterior view (A), lateral view (B), and time-of-flight source images (C), demonstrates stable findings.

## Discussion

The prevalence of dAVF has been estimated to be approximately 0.29 per 100,000 individuals, with only 1.2%-3.6% occurring in the anterior fossa [9,10]. The characteristics of anterior cranial fossa dAVF compared to other dAVFs include a higher incidence in males, a higher probability of onset due to intracranial hemorrhage, and the presence of varix [11]. In the present case, all these characteristics were applicable and the background of the patient was considered typical for an anterior cranial fossa dAVF. Although dAVF is often caused by acquired factors such as trauma or previous craniotomy, our patient had no such history.

Table 1 summarizes the 12 reported cases of concomitant moyamoya disease and dAVF, including the present case [4,5,[12], [13], [14], [15], [16], [17]]. The most common shunt site was the transverse-sigmoid sinus (TSS), which was involved in 6 cases. This finding is consistent with previous reports indicating that approximately 60% of dAVFs occur in the TSS [9,10,18]. In contrast, involvement of the anterior cranial fossa has been reported in only 1 previous case, making the present case the second reported instance. Although anterior cranial fossa dAVFs account for approximately 3% of all dAVFs, the number of reported cases associated with moyamoya disease remains far too small to support any statistical or anatomical inference. Therefore, the anatomical location in the present case should be regarded as a descriptive observation rather than evidence of a specific epidemiological trend.

**Table 1:** .

Patient no.	Study	Age	Sex	Presenting symptoms	Imaging finding on admission	Preceding/ symptomatic disease	Ditection of MMD and AVF	Suzuki stage	Side of AVF	Location of AVF	Drainer of AVF	Feeder of AVF	Borden classification of DAVF	Treatment for MMD	Treatment for AVF	Outome (mRS)
<Onset by MMD>																
1	Killory et al. [14]	44	M	Headache and tinnitus	IVH	MMD	Simultaneously	Rt 3 (unilateral)	Rt	TSS	TSS	OA, MMA, PAA, and AphA	I	STA-MCA bypass	TAE (ONYX) and TVE (coil)	0
2	Zaletel et al. [17]	71	M	Urinary retention and cognitive decline	SAH (bil frontal and parietal)	MMD	Simultaneously	Rt 4 and Lt 4	Rt	TSS	TSS	OA	I	No	Conservative	4
3	Hanaoka et al. [13].	45	F	Lt. hemiparesis	CI (R frontal lobe)	MMD	Delayed	Rt 3 and Lt 2	Lt	TSS	TSS	NA	II	STA-MCA bypass	TVE	NA
4	Feroze et al. [12]	51	F	Transient aphasia, extremity weakness, and headache	NA	MMD	Delayed	Bil 4	Bil	Pial vessel of convexity	Vein of Trolard	Branch of ICA	-	STA-MCA bypass	Conservative	NA
5	Koduri et al. [15]	14	F	Tonic–clonic seizure, facial droop, and hemibody weakness	CI (L hemispere and multifocal)	MMD	Delayed	Lt 3 and Rt NA	Bil	SS	SS	Bil MMA, OA, and muscular branches of VA	I	Bil pial synangoisis	Conservative	NA
6	Hou et al. [4]	47	M	Dizziness and gait disturbance	ICH/IVH, CI (cerebellum)	MMD	Delayed	Bil 4∼5	Lt	TSS	TSS and OS	MMA, OA,PMA	I	No (aspirin)	Conservative	0
7	Rao et al. [5]	63	M	Multiple infarction	Multiple recurrent CI, L hemisphere	MMD	Simultaneously	Lt 4 and Rt 1	Lt	Middle fossa (sphenoparietal)	Vein of Trolard	MMA	III	STA-MCA bypass and aspirin	Partial embolization + surgical ligation	NA
8	Rao et al. [5]	42	M	Transient headache	NA	(MMD)	Simultaneously	Rt 3 and Lt NA	Rt	**Anterior cranial fossa**	Frontal cortical vein to SSS	Opthalmic A	III	No (aspirin)	Conservative	NA
<Onset by DAVF>																
9	Liu et al. [16]	52	F	Proptosis and chemosis of right eye, and tinnitus	NA	DAVF	Simultaneously	Rt 3 (unilateral)	Rt	CS	SOV and IPS	MHT	I	No	TVE (faild) and spontaneous cure	0
10	Rao et al. [5]	57	M	Myelopathy	NA	DAVF	Simultaneously	Rt 3 and Lt NA	Lt	Cranio-cervical junction	Anterior/ posterior spinal vein	VA	III	No (aspirin)	Surgical ligation	NA
11	Rao et al. [5]	74	F	Vision loss and cognitive decline	Papilledema	DAVF	Simultaneously	Lt 4∼5 and Rt NA	Lt	TSS	TSS, SSS, and Labbe	MMA, tentrial A	II	No (aspirin)	TVE	NA
12	Present (case)	62	M	Headache and nausea	SAH/ICH	DAVF	Simultaneously	Rt 3 and Lt 3	Lt	**Anterior cranial fossa**	Frontal cortical vein to SSS	facial A and ethmoidal A	III	No	Surgical ligation	0

A, artery; APhA, ascending pharyngeal artery; AVF, arteriovenous fistula; Bil, bilateral; CI, cerebral infarction; CS, cavernous sinus; DAVF, dural arteriovnous fistula; F, female; ICA, internal carotid artery; ICH, intracerebral hemorrhage; IPS, inferior petrosal sinus; IVH, intraventricular hemorrhage; Labbe, vein of Labbe; Lt, left; M, male; MCA, middle cerebral artery; MHT, meningohypophyseal trunk; MMA, middle meningeal artery; MMD, moyamoya disease; mRS, modified Rankin Scale; NA, not applicable; OA, occipital artery; OS, occipital sinus; PAA, posterior auricular artery; PMA, posterior meningeal artery; Rt, right; SAH, subarachnoid hemorrhage; SOV, superior ophthalmic vein; SS, sigmoid sinus; SSS, superior sagittal sinus; STA, superficial temporal artery; TAE, transarterial embolization; TVE, transvenous embolization, TS, transverse sinus; TSS, transverse-sigmoid sinus; VA, vertebral artery.

The bold text is used to emphasize the AVF located at the same site as our present case.

Among the 12 reviewed cases, the mode of clinical presentation differed. Eight patients initially presented with symptoms related to moyamoya disease, whereas 4 patients presented with symptoms attributable to dAVF. In the former group, 4 cases were identified in which dAVF developed during the clinical course of moyamoya disease, including after surgical treatment. Conversely, in the latter group, moyamoya disease was diagnosed concurrently with the identification of dAVF. Although the limited number of cases and the lack of uniform long-term follow-up preclude definitive conclusions, these observations suggest that moyamoya disease may represent a preceding vascular condition in some cases of concomitant moyamoya disease and dAVF.

Treatment strategies were generally determined according to the mode of clinical presentation. Patients with cortical venous regurgitation (Borden types II and III) were frequently treated for dAVF, even in the presence of concomitant moyamoya disease. Among the reported cases, bypass surgery was performed in 5 patients presenting with cerebral infarction or intraventricular hemorrhage. In addition, antiplatelet therapy with aspirin was administered in 4 conservatively managed patients and in 1 patient who underwent bypass surgery.

The pathophysiological mechanism underlying the coexistence of moyamoya disease and dAVF remains unclear. It has been hypothesized that abnormal cerebral hemodynamics caused by progressive intracranial arterial stenosis may lead to cerebral ischemia, which in turn induces angiogenic responses in the dural vasculature, resulting in arteriovenous shunting [5,19]. Supporting this hypothesis, several molecular similarities have been reported between the 2 diseases. Increased expression of basic fibroblast growth factor and vascular endothelial growth factor has been demonstrated in the dura mater of patients with moyamoya disease [20,21], as well as in the dural arteries and venous sinus walls of patients with dAVF [22]. Although it remains uncertain whether these angiogenic or inflammatory mediators play a causal role or represent secondary compensatory responses, the presence of partially shared molecular features between the two conditions is noteworthy. In addition, conditions associated with moyamoya syndrome, particularly autoimmune disorders and prior radiation exposure, may induce inflammatory changes and coagulation abnormalities. These mechanisms may partially overlap with the pathophysiology and triggering factors proposed for dAVF formation.

In the present case, there was no clinical course suggestive of cerebral ischemia, and no imaging findings indicating ischemia were identified prior to the hemorrhagic presentation of the dAVF. These observations suggest that overt cortical ischemia may not be a mandatory prerequisite for dAVF formation in patients with moyamoya disease. Rather, even in the absence of clinically evident ischemia, chronic alterations in cerebral hemodynamics and venous drainage may contribute to the development of dural arteriovenous shunting.

It should also be noted that the primary vascular structures involved differ between the 2 diseases: moyamoya disease predominantly affects the arterial system, whereas dAVF is considered to arise mainly from pathological changes in veins or venous sinuses [19]. Despite this difference, most reported cases of concomitant moyamoya disease and dAVF, including the present case, have involved patients of East Asian ethnicity [5]. This observation raises the possibility of shared genetic susceptibility, such as variants in the RNF213 gene, which has been strongly associated with moyamoya disease [23]. The RNF213 variant, predominantly identified in East Asian populations, has been suggested to contribute to abnormal angiogenesis and endothelial dysfunction [24,25]. Although genetic testing was not performed in the present case, this potential association represents an important area for future investigation. Genetic testing for RNF213 variants was not available at our institution, representing an important limitation of the present study. Therefore, any potential genetic association in this case remains speculative. If shared genetic and molecular backgrounds were the primary mechanism underlying the coexistence of moyamoya disease and dAVF, the extremely small number of reported cases would be difficult to explain. Therefore, the possibility of additional unknown triggers, such as inflammatory processes, should be considered. Alternatively, the coexistence may be coincidental, or underrecognized in asymptomatic patients with moyamoya disease who have not undergone detailed angiographic evaluation.

## Conclusion

This illustrative case underscores the importance of careful follow-up and vascular screening in patients with moyamoya disease, given the rare coexistence of dAVF and moyamoya disease. Further accumulation of similar cases will be necessary to clarify the relationship between these two rare but clinically significant vascular disorders.

## Patient consent

A written informed consent was obtained from the patient for the publication of this case report.
